# Location, Location, Location: How Vascular Specialization Influences Hematopoietic Fates During Development

**DOI:** 10.3389/fcell.2020.602617

**Published:** 2020-11-13

**Authors:** Adam M. Heck, Takashi Ishida, Brandon Hadland

**Affiliations:** ^1^Clinical Research Division, Fred Hutchinson Cancer Research Center, Seattle, WA, United States; ^2^Department of Pediatrics, University of Washington School of Medicine, Seattle, WA, United States

**Keywords:** developmental hematopoiesis, hematopoietic stem cell, hemogenic endothelium, endothelial cell, vascular niches, aorta gonad mesonephros region, yolk sac, fetal liver

## Abstract

During embryonic development, sequential waves of hematopoiesis give rise to blood-forming cells with diverse lineage potentials and self-renewal properties. This process must accomplish two important yet divergent goals: the rapid generation of differentiated blood cells to meet the needs of the developing embryo and the production of a reservoir of hematopoietic stem cells to provide for life-long hematopoiesis in the adult. Vascular beds in distinct anatomical sites of extraembryonic tissues and the embryo proper provide the necessary conditions to support these divergent objectives, suggesting a critical role for specialized vascular niche cells in regulating disparate blood cell fates during development. In this review, we will examine the current understanding of how organ- and stage-specific vascular niche specialization contributes to the development of the hematopoietic system.

## Introduction

The blood and the vascular systems are intimately connected in their evolutionary and developmental origins. Endothelial cells (ECs), essential to the establishment of a closed circulatory system, are hypothesized to have evolved from amoebocytes, ancestral phagocytic blood cells (Monahan-Earley et al., 2013). It is therefore not surprising that, during embryonic development, the first hematopoietic cells and ECs arise together in close association within the blood islands of the extraembryonic yolk sac and are thought to share a common mesodermal precursor cell type referred to as the hemangioblast (Choi et al., 1998; Huber et al., 2004). The fates of blood cells and ECs remain intertwined throughout development. Interconversion between endothelial and hematopoietic cells, referred to as the endothelial to hematopoietic transition, is an important feature of embryonic development that is responsible for sequential waves of hematopoiesis in the developing vasculature of multiple tissues (yolk sac, placenta, aorta, head, heart, and, intriguingly, perhaps even in the fetal and newborn bone marrow) (Nishikawa et al., 2000; Li et al., 2005, 2012; Gordon-Keylock et al., 2013; Nakano et al., 2013; Frame et al., 2016; Yzaguirre and Speck, 2016; Kasaai et al., 2017; Shigeta et al., 2019; Yvernogeau et al., 2019). In the reverse direction, this interconversion is also manifested by the contribution of yolk sac-derived hematopoietic progenitors to ECs in the developing vasculature of various organs (Plein et al., 2018). Plasticity between endothelial and hematopoietic fates is a feature that is also reflected in adult cells. This is highlighted by the fact that hematopoietic stem cells (HSCs) in the adult bone marrow are distinguished from more differentiated blood cells by genes that significantly overlap with those expressed by ECs (perhaps some of which are necessary to mediate adhesive and signaling interactions between HSCs and ECs to support HSC maintenance in the marrow vascular niche) (Chen et al., 2002; Balazs et al., 2006; Kent et al., 2009; Gazit et al., 2014; Carrelha et al., 2018). Furthermore, the ability to convert adult ECs into functional HSCs by the induced expression of only four transcription factors also attests to the close relationship of these two cell types (Sandler et al., 2014; Lis et al., 2017).

The signaling pathways and the transcriptional programs that specify and distinguish endothelial and hematopoietic fates have been the subject of much study and are extensively reviewed elsewhere (Ciau-Uitz et al., 2014; Frame et al., 2017; Perlin et al., 2017a; Dzierzak and Bigas, 2018; Gao et al., 2018; Menegatti et al., 2019; Slukvin and Uenishi, 2019). Even once their fates are distinctly specified, however, endothelial and hematopoietic cells continue to share close spatial relationships, forming specialized microenvironments in which cross-communication is an essential aspect of hematopoietic maintenance and differentiation throughout the course of development and adulthood. In the context of the adult bone marrow, this concept is supported by numerous studies on the contributions of various vascular endothelial subsets to hematopoietic niche compartments that support HSC self-renewal and differentiation to diverse blood cell lineages, which is reviewed elsewhere (Crane et al., 2017; Pinho and Frenette, 2019). In the embryo, where the opposing processes of rapid blood cell differentiation and proliferative HSC self-renewal are both essential, the primary sites of hematopoiesis are dynamically shifting across development. In this context, the complexity of vascular niche–hematopoietic interactions has not been as extensively characterized. However, many recent, intriguing studies have pointed to the important role of vascular niche specialization, as regulated by diverse inputs such as biomechanical forces downstream of blood flow dynamics, to the control of disparate blood cell fates throughout embryonic development. Furthermore, these studies have begun to reveal how unique cell intrinsic properties of hemogenic populations at various stages of development integrate with signals from different vascular niches to define distinct hematopoietic potentials. Here we will attempt to synthesize the current knowledge of the major anatomical sites of hematopoiesis throughout development, highlighting the role of specialized vascular microenvironments at each site in orchestrating unique aspects of hematopoiesis responsible for producing diverse types of embryonic blood cells and HSCs (Table 1). We will also provide some perspectives on missing gaps in the current understanding of vascular niche regulation of hematopoietic fates during development and examine how this knowledge may advance therapeutic applications in hematopoietic cell transplantation and other cellular therapies.

**TABLE 1:** Hematopoietic vascular niches throughout development.

Organ/tissue	Vascular niche	Hematopoietic activity	Types of niche signals/pathways
Yolk sac	Blood islands	Generation and maturation of primitive progenitors (erythroid, macrophage, megakaryocyte)	Canonical Wnt, Notch (inhibitory), retinoic acid (dynamic), Vcam1/integrins (inhibitory)
	Early vascular plexus	Generation of EMPs	Wnt, cytokines
	Early arterial vessels	Generation of LPs, LMPs, MPPs	Notch
Heart	Endocardium	Generation EMPs	
P-Sp/aorta–gonad–mesonephros regions	Aorta	Generation of LMPs, MPPs, HSCs	Wnt (dynamic), Notch (dynamic), retinoic acid, fluid shear stress, cyclic stretch, chemokines (Cxcl12), catecholamines, pro-inflammatory signals, hyaluronan, extracellular matrix/integrins
Extraembryonic vessels	Vitelline artery	Generation of HSCs	
	Umbilical artery	Generation of HSCs	
Head	Cerebrovascular endothelial cells	Generation of HSCs	
Placenta	Chorioallantoic vessels	Generation of MPPs, HSCs	EC, trophoblast, pericyte, and stromal cell-derived cytokines/chemokines
	Vascular labyrinths	Expansion of MPPs, HSCs	
Fetal liver (caudal hematopoietic tissue)	Sinusoids	Recruitment and expansion of HSCs, proliferation and differentiation of EMPs, LMPs, MPPs	EC-derived chemokines/cytokines, periostin/matricellular proteins, docosahexaenoic acid– free fatty acid/lipids
	Portal vessels/pericytes	Expansion of HSCs	Hemodynamic blood flow, pericyte-derived cytokines/chemokines
Fetal bone marrow	Sinusoids	Generation of MPPs, HSCs	

*EMPs, erythromyeloid progenitors; LPs, lymphoid progenitors; LMPs, lymphoid–myeloid progenitors; MPPs, multipotent progenitors.*

## HSC-Independent Hematopoietic Progenitors in the Yolk Sac Vascular Niche

### Primitive Blood Cells Develop Concurrently With Endothelium in the Yolk Sac Blood Islands

The first mammalian blood cells arise following primitive streak formation from mesodermal precursors that migrate to the yolk sac to form structures known as blood islands. The organization of blood islands, in which blood cells form in aggregates surrounded by ECs, led to the initial hypothesis that blood and ECs arise from common clonal mesodermal precursors, referred to as hemangioblasts. The existence of clonal hemangioblasts has been demonstrated *ex vivo* in the context of differentiating murine embryonic stem cells (ESCs) (Choi et al., 1998) as well as during gastrulation *in vivo* (Huber et al., 2004). However, more recently, studies have suggested a polyclonal origin for blood islands and demonstrated that asynchronous waves of mesodermal derivatives from the primitive streak differentially contribute to initial endothelial and hematopoietic fates (Furuta et al., 2006; Ueno and Weissman, 2006; Padrón-Barthe et al., 2014).

In contrast to adult hematopoiesis, where essentially all circulating blood cells are derived from HSCs residing in the bone marrow niche, the first blood cells during embryonic development are generated without transitioning through an HSC state. These initial phases are thus referred to as HSC-independent waves of hematopoiesis (Palis, 2016a; Dzierzak and Bigas, 2018; Hadland and Yoshimoto, 2018). The first HSC-independent wave, which occurs in the yolk sac blood islands, is termed the primitive wave since it generates unique, initially nucleated “primitive” erythrocytes, providing the first circulating red blood cells of the embryo (Moore and Metcalf, 1970; Palis et al., 1999, 2010; Tober et al., 2007). The primitive wave, which also includes other distinctive lineages of primitive macrophages and megakaryocytes, gives rise to a rapid and transient burst of hematopoietic activity shortly after murine embryonic day 7 (E7) (Palis et al., 1999; Tober et al., 2007). The ability to rapidly generate blood cells directly from mesodermal precursors (hemangioblasts) shortly after primitive streak formation is likely essential to meet the immediate needs of the developing embryo. Interestingly, though the hematopoietic progeny of this primitive wave have largely been considered transient, the descendants of HSC-independent yolk sac macrophages from this wave have been shown to persist into adulthood as tissue-resident macrophages, providing functions in some adult organs such as the microglia of the brain (Ginhoux et al., 2010; Ginhoux and Guilliams, 2016).

Several exogenous, niche-derived signals have been shown to support the sequential aspects of the primitive wave of hematopoiesis in the yolk sac, coordinating its rapid onset and subsequent termination. The yolk sac visceral endoderm is a source of initial key signals, including VEGF and Indian Hedgehog, that promote the migration and induction of mesodermal cells from the primitive streak to form endothelial and hematopoietic progeny in the blood islands (Shalaby et al., 1995, 1997; Baron, 2001; Byrd et al., 2002; Damert et al., 2002); however, explant studies demonstrated that erythropoiesis can proceed from the yolk sac mesoderm in the absence of visceral endoderm or an endothelial cell network, suggesting that the cell-intrinsic hematopoietic potential of mesodermal precursors is already established upon their migration to the yolk sac (Palis et al., 1995). Studies using differentiation of murine ESCs into “embryoid bodies” *in vitro*, which provide a robust model for the initial yolk sac waves of hematopoiesis including recapitulation of blood island-like structures producing primitive hematopoietic progenitors and ECs (Doetschman et al., 1985; Risau et al., 1988; Wang et al., 1992; Bautch et al., 1996), have also provided insight into essential signals for early hematopoiesis. For example, this *in vitro* model demonstrated important roles for BMP4 and VEGF pathways in establishing the hematopoietic potential of mesodermal precursors at the level of the hemangioblast, which is characterized by the expression of the VEGF receptor Flk1 (Choi et al., 1998; Park et al., 2004; Nostro et al., 2008). Specifically, BMP4 promotes the generation of Flk1^+^ hemangioblasts with endothelial and hematopoietic potential during mesoderm formation, whereas VEGF supports the subsequent specification, expansion, and/or survival of progenitors with hematopoietic colony-forming capacity *in vitro*.

Interactions between key developmental pathways involving Notch and Wnt/β-catenin signaling have also been shown to be important in regulating primitive hematopoietic fates following mesoderm induction. The Notch pathway is activated by intercellular binding between one of a family of transmembrane Notch ligands expressed on a sending cell and a Notch receptor expressed on a receiving cell. This interaction leads to the proteolytic cleavage of the Notch intracellular domain and downstream transcriptional activation of Notch target genes (reviewed in Kopan and Ilagan, 2009). Conversely, activation of Wnt signaling occurs when a Wnt ligand family member, most of which are secreted, binds to a Frizzled receptor, triggering downstream Wnt signaling responses that are canonical or non-canonical based on their dependence or independence on β-catenin, respectively (reviewed in de Jaime-Soguero et al., 2018). In the context of primitive hematopoiesis, transient canonical Wnt/β-catenin signaling was shown to promote primitive erythroid fate from hemangioblast-like precursors derived from the mesoderm of differentiating murine ESCs. In contrast, activation of Notch signaling negatively regulates primitive erythropoiesis by inducing the expression of a number of secreted Wnt pathway inhibitors including Sfrp1, Sfrp5, Wnt5a (a non-canonical Wnt ligand that can have context-dependent inhibitory effects on canonical Wnt/β-catenin signaling), and Dkk1 (Cheng et al., 2008). Notch signaling is transiently blocked by the expression of an endogenous inhibitory protein, Numb, to enable coordinated Wnt-dependent primitive erythroid differentiation from hemangioblasts. A similar role for Notch/Numb interactions has also been shown to regulate primitive erythrocyte maturation in the zebrafish embryo, suggesting a conserved function in this process (Bresciani et al., 2010). Altogether, these studies suggest that the rapid and transient nature of primitive erythroid differentiation from hemangioblasts may in part be tightly regulated by the opposing interactions of the Wnt and the Notch pathways. However, it remains unclear precisely which ligands are responsible for sequentially activating the Wnt and the Notch pathways during their interactions in primitive erythropoiesis and whether these ligands are expressed by developing ECs that are in close proximity to the developing primitive erythrocytes of the blood islands or are produced by another cell type in the yolk sac niche.

Other dynamic signaling pathways have also shown to contribute to the transient emergence of primitive erythrocytes in the blood islands. For example, vitamin-A derived retinoic acid signals function upstream of BMP4 to promote the survival of mesodermal precursors that contribute to blood island-derived primitive erythrocytes in avian explant models of yolk sac development (Ghatpande et al., 2002). Intriguingly, retinoic acid also has an inhibitory role at the later stages of primitive hematopoiesis, acting as a negative regulator upstream of hematopoietic transcription factor *Scl* in both zebrafish and mouse embryo/ESC systems (de Jong et al., 2010). Additionally, vascular cell adhesion molecule-1 (Vcam-1), whose expression in mesenchymal stromal cells is regulated by the microRNA miR-126, was shown to accelerate the maturation of primitive erythrocytes in an ESC-derived embryoid body model via activation of a Src-family kinase, thus facilitating the termination of the primitive erythroid progenitor wave (Sturgeon et al., 2012). This effect is mediated by interactions between Vcam-1 expressed on the mesenchymal cells and an integrin receptor, potentially VLA-4 (α4/β1-integrin), which is expressed on primitive erythrocytes and is later required for their enucleation via interactions with Vcam-1-expressing macrophages in the fetal liver (Palis, 2016b). ESC-derived Vcam-1^+^ cells were shown to have the potential to generate multiple mesenchymal lineages *in vitro*, and a comparable Vcam-1^+^ population, distinct from both hematopoietic and ECs, was also identified in the yolk sac, suggesting a similar role for Vcam-1-expressing mesenchymal cells *in vivo*.

Altogether, the studies discussed above suggest that multiple interacting signals from unique niche populations of the early yolk sac are integrated to regulate the transitory nature of the first wave of hematopoiesis from blood islands. Specifically, by coordinating the rapid emergence and cessation of primitive hematopoietic progenitors, these signals contribute to generating a rapid burst of circulating erythrocytes necessary for oxygen delivery in the developing embryo at the onset of circulation, which initiates shortly after the completion of primitive hematopoiesis, around E8.25 in murine development. A number of interesting questions regarding how the blood island vascular niche regulates the primitive wave of hematopoiesis remain to be addressed. For example, the nature of signaling interactions between the tightly associated hematopoietic and endothelial cells of the blood islands and how these interactions contribute to different cell fates remain largely unexplored. Furthermore, how the distinct lineages of primitive erythrocytes, macrophages, and megakaryocytes arise from hematopoietic progenitors in the blood islands and whether different niche-derived signals regulate these disparate fates are yet unclear. Importantly, such information could have relevance to understanding the origin of tissue-resident macrophages with potential therapeutic value given their role in tissue homeostasis in adult organs such as the brain.

### Erythromyeloid Progenitors Arise From Hemogenic Endothelium in Vessels Lacking Arterial–Venous Specification in the Early Yolk Sac and Other Tissues

Following the initial wave of primitive blood progenitors arising in yolk sac blood islands, a second wave of HSC-independent hematopoietic progenitors is detected around E8. This wave produces erythro-myeloid progenitors (EMPs) that are largely restricted to erythroid, megakaryocyte, and myeloid lineages, though some studies have suggested rare B-lymphocyte potential as well as NK-cell potential (McGrath et al., 2015; Dege et al., 2020). EMPs are distinguished from the primitive wave by their generation of adult-like enucleated erythrocytes with a distinct globin gene expression profile, as well as by their ability to generate granulocytes in addition to macrophages (McGrath and Palis, 2008). In contrast to the primitive progenitors that arise *in situ* in the blood islands, EMPs arise as the yolk sac ECs begin to reorganize into a vascular plexus, suggesting that this process may be distinctly important during the EMP wave of hematopoiesis. Indeed, recent studies observed that, within the nascent yolk sac vessels, EMPs arise from a specialized subset of ECs termed hemogenic endothelium (HE) and also contribute to the process of yolk sac vascular remodeling (Chen et al., 2011; Padrón-Barthe et al., 2014; Frame et al., 2016; Kasaai et al., 2017). HE initially possess morphologic and transcriptional characteristics of vascular ECs before undergoing a transition to hematopoietic progenitor cells, a process commonly referred to as the endothelial to hematopoietic transition, which is orchestrated by hematopoietic-specifying transcription factors primarily driven by *Runx1* (Sugiyama et al., 2003; Frame et al., 2016; Kasaai et al., 2017; Yzaguirre et al., 2017). Interestingly, the unique HE that produces EMPs reflects the transcriptional and the phenotypic properties of endothelium in the yolk sac vascular plexus at this stage, which largely lacks arterial specification (Chen et al., 2011; Frame et al., 2016; Gao et al., 2018; Yzaguirre et al., 2018). Furthermore, EMPs can arise in the absence of hemodynamic forces from blood flow, which initiates shortly after E8 and is essential for later arterial–venous specialization of yolk sac vessels (le Noble et al., 2004; Lucitti et al., 2007; Lux et al., 2008). Lineage tracing studies using EC-specific genes including *Nrp2* (expressed in non-arterial ECs) and *Lyve1* (expressed in yolk sac ECs and lymphatic vascular beds) support the notion that EMPs are generated from HE unique from precursors of both the earlier primitive wave of hematopoiesis in the yolk sac and from later waves of intra-embryonic hematopoiesis giving rise to HSCs (Wiszniak et al., 2015; Lee et al., 2016).

Similar to the primitive wave, the development of EMPs is dependent on canonical Wnt/β-catenin signals from the vascular niche, though the exact mechanism of Wnt pathway activation and the source of Wnt ligands are unclear. However, in contrast to the primitive wave, there does not seem to be an inhibitory role for Notch signaling in the development of EMPs (Hadland et al., 2004; Cheng et al., 2008; Frame et al., 2016). The initial proliferation of EMPs after their formation in the yolk sac vessels is dependent on signaling through c-kit, the receptor for stem cell factor (SCF) (Azzoni et al., 2018). To this end, yolk-sac derived endothelial lines have been shown to produce several hematopoietic-supportive cytokines including SCF, FLT-3 ligand, macrophage colony-stimulating factor, leukemia-inhibitory factor, and interleukin-6, suggesting a pro-hematopoietic environment in the yolk sac vascular niche that probably contributes to the initial formation, survival, and expansion of EMPs (Fennie et al., 1995). Despite this environment, the yolk sac does not appear to be an effective niche for differentiation of mature blood cells from EMPs (Rampon and Huber, 2003). This is in contrast to the rapid maturation of primitive erythrocytes that occur in the blood islands during the primitive wave. Instead, after circulation is established, EMPs migrate to the fetal liver, where they take residence, continue to divide and differentiate, and generate a second burst of functional blood cells for the maturing embryo (Palis, 2016a).

Although the yolk sac serves as the initial and the primary site for the production of HSC-independent waves of hematopoiesis, other sites in the embryo proper, such as the heart, appear to transiently produce HSC-independent, EMP-like progenitors from endocardial ECs, which, similar to yolk sac HE, lack arterial–venous specialization (Nakano et al., 2013). Moreover, ectopic expression of Runx1 was found to be sufficient to induce EMPs from non-hemogenic endothelium at multiple sites in the early yolk sac and embryo proper (including the dorsal aorta and the heart), but only during a limited window of development (murine E7.5 to E8.5), before circulation or the arterial–venous specialization of ECs is well-established (Yzaguirre et al., 2018). These findings suggest that endothelial maturation and specialization, integrated with hemodynamic changes in the vascular niche microenvironment, may be important in limiting the window of EMP production during early embryonic development. However, further studies will be necessary to test this hypothesis. Similar to the primitive wave, the EMP wave is presumed to generate transient blood cells that support the needs of the growing embryo; however, recent lineage tracing studies support the notion that myeloid progenitors from this wave also contribute to tissue-resident macrophages in various adult organs (reviewed in Palis, 2016a).

### The First Lymphoid Progenitors Arise From Hemogenic Endothelium in Nascent Arterial Vessels of the Yolk Sac and Early Embryo

In addition to generating EMP, the yolk sac also serves as an initial source of T and B lymphoid progenitors and lymphoid–myeloid progenitors (LMPs), which emerge essentially simultaneously in the developing dorsal aorta in the para-aortic splanchnopleura (P-Sp, which later gives rise to the aorta–gonad–mesonephros regions, or AGM). Similar to the primitive and EMP waves, these progenitors are also believed to initially arise independently of HSCs (Godin et al., 1993; Yoshimoto et al., 2011, 2012; Böiers et al., 2013; Kobayashi M. et al., 2014; Lin et al., 2019). Beyond the scope of this review, there is growing evidence for the contribution of these earliest waves of lymphocytes to unique innate-like immune cell populations distinct from those arising later from HSCs, some of which also persist into adulthood and contribute to aspects of immunity throughout life (reviewed in Hadland and Yoshimoto, 2018). The emergence of these lymphoid-competent progenitors corresponds with the onset of arterial–venous specialization and circulation in the yolk sac vasculature. In contrast to EMPs, these lymphoid progenitors arise from HE with arterial characteristics (Yzaguirre and Speck, 2016), suggesting important roles for arterial endothelial niche and hemodynamic forces in the specification of hematopoietic cells with lymphoid potential. Supporting this association, Notch1 signaling, which plays an essential role in arterial endothelial specification (Lawson et al., 2001; Corada et al., 2014) and stabilization of arterial transcriptional programs in response to blood flow (Lawson et al., 2001; le Noble et al., 2004; Hwa et al., 2017; Weijts et al., 2018), is required for multilineage hematopoiesis and HSC development *in vivo* (Kumano et al., 2003; Hadland et al., 2004) and has been shown to play a role in conferring lymphocyte potential to nascent stem/progenitor cells derived during the differentiation of pluripotent stem cells (Lu et al., 2016; Uenishi et al., 2018). The role of Notch signaling and blood flow hemodynamics in the emergence of multilineage hematopoiesis and HSCs will be discussed in more detail in the following section, “HSC Development in the Arterial Vascular Niche” of this review. Much remains to be uncovered about how the vascular niche of the yolk sac and early aorta regulate the emergence of this wave of HSC-independent lymphocyte progenitors. For example, beyond a role for Notch, it is unclear how other signaling pathways may be involved in promoting the emergence of these first lymphoid-competent progenitors and how these signals may be distinct from those required for the emergence of HSCs, which overlap in the location and timing of their formation from HE in arterial vessels. Given the role of these early lymphocytes in establishing initial immunity during development and the persistence of some HSC-independent lymphocyte lineages into adulthood, this knowledge could have important implications in understanding how the immune system develops and how it is dysregulated in the context of autoimmunity and other immune disorders.

## HSC Development in the Arterial Vascular Niche

**FIGURE 1:**
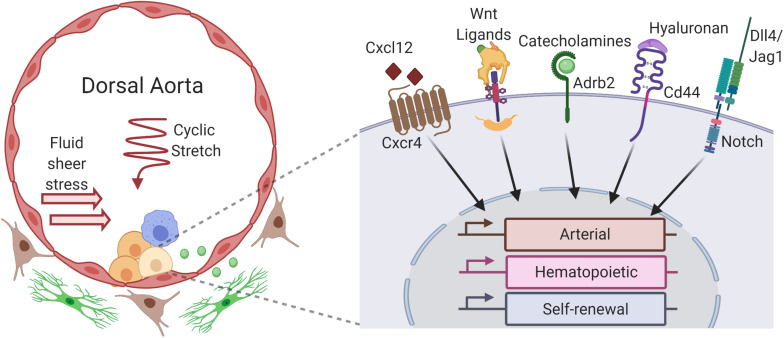
Niche factors of the aorta–gonad–mesonephros region (AGM) influence hematopoietic stem cell (HSC) gene expression and development. Within the dorsal aorta of the AGM, hemogenic endothelial cells undergo an endothelial to hematopoietic transition to give rise to HSCs (light yellow) and other hematopoietic progenitors (yellow), forming hematopoietic clusters that line the aortic lumen. HSC development is influenced by numerous cells that constitute the AGM vascular niche, including neighboring hematopoietic cells in the clusters (yellow), yolk sac-derived macrophages (blue) that migrate to the aorta, underlying arterial ECs (red) of the aortic lumen, subluminal sympathoadrenal cells (green), and other stromal cells (brown) in the mesenchyme surrounding the aorta. Autocrine, juxtracrine, and paracrine signals from these niche cells integrate with hemodynamic forces like fluid sheer stress and cyclic stretch to activate receptors and downstream signaling pathways within developing HSCs, including Cxcr4, Wnt/β-catenin, β_2_-adrenergic receptor (Adbr2), CD44, and Notch, among others. These pathways modulate the dynamic expression of arterial, hematopoietic, and self-renewal gene expression programs which drive the emergence of HSCs from hemogenic endothelium (created with BioRender.com).

As described in the preceding section, “HSC-Independent Hematopoietic Progenitors in the Yolk Sac Vascular Niche,” the HSC-independent waves of hematopoiesis that are critical for the initial production of blood cells in the developing embryo occur predominantly in the yolk sac, with contributions from intraembryonic vascular sites as well (Palis, 2016a). With exceptions as noted above, the majority of cells from these populations are transient and eventually replaced by the HSC-dependent wave of hematopoiesis, which occurs exclusively in arterial vessels later in development (de Bruijn et al., 2000; Gordon-Keylock et al., 2013). During this wave, a unique population of arterial HE undergoes the endothelial to hematopoietic transition, ultimately giving rise to HSCs that are defined by their capacity to provide long-term, multilineage engraftment in conditioned adult recipients. Functional HSC activity is detected shortly after E10.5 in multiple arterial vessels, including the dorsal aorta of the AGM region and the extraembryonic vitelline and umbilical arteries. Herein, we describe some of the major pathways that are involved in this process and highlight how the unique components of the arterial vascular niche influence the activation of these pathways to regulate HSC development (Figure 1).

### The Role of Notch Signaling in HSC Development

While Notch signaling appears to have an inhibitory role in primitive hematopoiesis and is expendable in the EMP wave, it is essential for HSC development. Specifically, the deletion of *Notch1* in murine embryos abrogates HSC generation in the P-Sp/AGM, and experiments involving murine embryos chimeric for *Notch1*-deficient cells demonstrated a cell-autonomous requirement for Notch1 in establishing long-term, multilineage hematopoiesis (Kumano et al., 2003; Hadland et al., 2004). While Notch1 is expressed in the hematopoietic clusters that line the aortic lumen, both Notch1 and Notch4 receptors are expressed by ECs of the dorsal aorta and demonstrate partially overlapping functions in arterial vascular development (Krebs et al., 2000; Robert-Moreno et al., 2005, 2008). Moreover, Notch ligands Dll4, Jagged1, and Jagged2 are expressed heterogeneously in aortic ECs and hematopoietic clusters (Robert-Moreno et al., 2005, 2008). Interestingly, loss-of-function studies demonstrated that Jagged1 plays an important role in hematopoietic specification in the AGM, without affecting the integrity of the arterial vessels, whereas Dll4 is required for earlier arterial endothelial specification and vasculogenesis (Duarte et al., 2004; Robert-Moreno et al., 2008). These early genetic studies suggested sequential requirements for the Notch pathway, mediated by differential interactions with Dll4 and Jagged1, during the specification of arterial ECs and the formation of HSCs from HE in AGM.

Similar to the first lymphoid progenitors in the yolk sac, which seem to arise from arterial vessels, recent studies have demonstrated that HE with multilineage hematopoietic and HSC potential first acquire the markers and the transcriptional signatures of the arterial endothelial program (Hadland et al., 2017; Uenishi et al., 2018; Slukvin and Uenishi, 2019; Hou et al., 2020). Arterial fate determination is a hallmark function of the Notch pathway, which is underscored by multiple loss-of-function studies for various Notch pathway members demonstrating defects in arterial specification and varying degrees of vascular malformation (Xue et al., 1999; Krebs et al., 2000, 2004; Duarte et al., 2004; North et al., 2009; Sorensen et al., 2009). At a molecular level, Notch signaling induces the expression of several key arterial transcription factors including *Hey1*, *Hey2*, and *Sox17* (Fischer et al., 2004; Corada et al., 2013; Sacilotto et al., 2013; Chiang et al., 2017). Intriguingly, Sox17 also works upstream of Notch to promote its activity, suggesting that Sox17 and Notch may participate in a feed-forward loop which drives the acquisition and the maintenance of arterial identity (Corada et al., 2013; Chiang et al., 2017). Although the relationship between HE and non-hemogenic arterial ECs has been debated (Ditadi et al., 2015; Gama-Norton et al., 2015), recent evidence using clonal analysis of lineage potential *in vitro* combined with single-cell RNA sequencing suggests that HSC-competent HE and non-hemogenic arterial ECs diverge from a common arterial-like EC precursor shortly after E8 (Hou et al., 2020). Moreover, another recent study used single-cell RNA sequencing and ATAC-seq (assay for transposase-accessible chromatin sequencing) to identify a “pre-HE” state in a subset of arterial ECs with the potential to give rise to HSCs and LMPs in murine embryonic caudal arteries (dorsal aorta, umbilical artery, and vitelline artery) (Zhu et al., 2020). Specifically, pre-HE were shown to express arterial EC-specific and Notch pathway target genes (e.g., *Hey2*, *Sox17*) and simultaneously exhibit a chromatin accessibility profile indicating susceptibility to hematopoietic-specifying transcription factors. Altogether, these studies suggest that the initial function of Notch1 signaling upstream of the generation of HSCs and lymphoid-competent progenitors likely reflects its requirement for arterial endothelial specification.

Following arterial specification, however, HE must then activate hematopoietic gene programs during the endothelial to hematopoietic transition, a process which is intrinsically linked to loss of endothelial fate and a reduction in Notch signaling (Gama-Norton et al., 2015; Lizama et al., 2015). Specifically, a reduction in Notch activity following the transition to HE results in the loss of arterial programming and enables hematopoietic fate acquisition that is required for HSC emergence. While cell-intrinsic reduction of Notch1 receptor expression may play a role in this process (Zhang et al., 2015, 2017), changes in Notch activity levels and function may also be due to differences in Notch receptor–ligand interactions over the course of HSC development in the arterial vascular niche of the AGM. Specifically, Dll4 may promote the initial high Notch activity required for arterial programming, whereas Jagged1 facilitates lower Notch signaling later on in the process required for hematopoietic fate (Gama-Norton et al., 2015). Consistent with this notion, recent studies have demonstrated a specific inhibitory role for Dll4 in the recruitment of HE to nascent hematopoietic clusters forming in the AGM (Porcheri et al., 2020), presumably by sustaining high Notch signal activity in HE, thus maintaining the expression of arterial genes such as *Hey1* and *Hey2* and preventing the endothelial to hematopoietic transition. On the other hand, perhaps as a function of lower Notch signal strength mediated by interactions with Jagged1, the Notch pathway also promotes the downstream activation of genes required for HSC fate, including *Runx1*, *Hes1/5*, *Gata2*, and *Cdca7* (Burns et al., 2005; Robert-Moreno et al., 2005; Nakagawa et al., 2006; Guiu et al., 2013, 2014). Furthermore, although the temporal window of Notch dependency seems to wane in the latter stages of HSC development in the AGM (Souilhol et al., 2016), some level of Notch signaling seems to be required for subsequent HSC expansion and maintenance *in vitro* and *in vivo* (Gerhardt et al., 2014; Hadland et al., 2015). Altogether, these findings underscore the concept that changes in Notch receptor–ligand interactions likely play a crucial role in regulating dynamic Notch signaling activity to facilitate the different phases of HSC development in the AGM vascular niche. However, future studies are needed to fully characterize the mechanisms by which the precise timing and tuning of Notch pathway activity are orchestrated to regulate this process, knowledge of which will be essential for efforts to recapitulate HSC development from pluripotent stem cells *in vitro*.

### The Role of Wnt Signaling in HSC Development

Just as the canonical Wnt/β-catenin pathway plays an essential role in yolk sac primitive and EMP waves of hematopoiesis, it is also crucial for HSC emergence in the AGM. Recent studies suggest that temporal changes in Wnt signaling may have a profound impact on HSC production. Wnt signaling pathway components Disheveled, TCF/LEF, and β-catenin, as well as multiple Wnt ligands and receptors, are expressed in ECs in the dorsal aorta (E9.5–11.5) (Orelio and Dzierzak, 2003; Corrigan et al., 2009; Ruiz-Herguido et al., 2012). Moreover, detectable nuclear localization of β-catenin, a hallmark of canonical Wnt pathway activation, is restricted to a small subset of aortic ECs near the base of, but not within, intra-aortic hematopoietic clusters (Ruiz-Herguido et al., 2012; Chanda et al., 2013), suggesting that Wnt signaling may influence HSC emergence from HE during this time. Supporting this notion, the exogenous activation of the Wnt pathway, via a GSK3β small-molecule inhibitor, at E10.5 enhances HSC production from the AGM whereas suppression of Wnt activity reduces HSC emergence (Ruiz-Herguido et al., 2012). In contrast, Chanda and colleagues observed that the downregulation of Wnt signaling at E11.5, either exogenously via pharmacological inhibition or endogenously through mechanisms downstream of retinoic acid signaling, is crucial for HSC development (Chanda et al., 2013). Although these findings appear contradictory, they suggest that, much like the Notch pathway activation, precise temporal changes in Wnt activity are necessary during HSC development. Specifically, these combined studies suggest a model in which canonical Wnt pathway activity is transiently required for the generation of HSC-competent HE but must subsequently be downregulated to promote proper HSC development. This proposed model provides a rationale for the observation that inactivation of β-catenin in VE-Cadherin-expressing EC/HE, but not in Vav1-expressing hematopoietic cells, abrogates HSC generation (Ruiz-Herguido et al., 2012).

To date, the specific gene programs that are dynamically activated by Wnt signaling during HE specification and HSC emergence remain poorly characterized. However, several studies that have demonstrated a role for Wnt signaling in the formation of the vascular niche may provide insight. Loss-of-function mutations of β-catenin result in defective vascular patterns in several embryonic regions, including the umbilical and vitelline arteries, and ultimately lead to lethality by E11.5 (Cattelino et al., 2003). Additionally, arterialization is dependent on crosstalk between Wnt and Notch pathways. Active β-catenin promotes the expression of arterial genes *Hey1*, *Hey2*, and *EphrinB2* while also directly stimulating the expression of Notch ligand Dll4, which in turn activates the Notch pathway (Corada et al., 2010). Thus, Wnt signaling may act upstream or in parallel with Notch signaling to promote the acquisition of arterial gene programs during the specification of arterial HE in the aorta and other arterial vessels where HSCs originate. However, direct evidence for interactions between these pathways during HSC development from arterial HE is yet lacking, and thus future studies are needed to understand the precise role of Wnt signaling during this process and whether a direct interaction with the Notch pathway is essential.

The source of ligands regulating the dynamic activation of the Wnt pathway during HSC development also remains to be determined. In zebrafish, the secretion of Wnt ligands like Wnt16 and Wnt9a by somite-mesoderm cells has been shown to be crucial for proper HE specification during the formation of the aorta (Clements et al., 2011; Grainger et al., 2016). *In vitro* coculture studies have also provided evidence that niche stromal cells derived from the AGM are able to influence the Wnt signaling pathway by secretion of Wnt ligands. For example, soluble Wnt5a, a non-canonical Wnt ligand that can have context-dependent activating or inhibitory effects on non-canonical Wnt/β-catenin signaling, was found to maintain repopulating HSCs *in vitro*, and its expression was observed to be a differentiating factor between supportive and non-supportive AGM-derived stromal cell lines (Buckley et al., 2011). However, it remains unclear how these stromal cell lines relate to endogenous niche cells in the AGM and whether Wnt5a plays an essential role in HSC formation from HE *in vivo*. Future studies addressing topics like these will provide clarity as to the precise role Wnt signaling plays during HSC emergence in the AGM.

### The Role of Chemokines in HSC Development

In addition to mediating cell-to-cell signals such as through the Notch pathway, the arterial ECs of the AGM vascular niche also secrete chemokines, like CXCL12, which facilitate various aspects of HSC development through paracrine and/or autocrine signaling to HE. The interaction of CXCL12 with its receptor CXCR4 has been well-studied in the context of the adult bone marrow where it is involved in HSC homing and quiescence (Zhang et al., 2016; Reid et al., 2018). Less is known about the potential role of CXCL12/CXCR4 interactions during HSC development in the AGM; however, several studies have begun to reveal interesting insights. In zebrafish, the CXCL12 homolog (cxcl12b) is expressed in a subpopulation of arterial ECs in the dorsal aorta that are distributed in a salt-and-pepper manner (Cha et al., 2012; Nguyen et al., 2014). Several independent fate mapping strategies revealed that this subpopulation of ECs is derived from a central somite region termed the “endotome.” These endotome-derived ECs migrate to the aorta and help induce HSC potential from HE that are derived from lateral plate/splanchnic mesoderm. Consistent with this model, loss of cxcl12b function in zebrafish, either genetically or chemically, reduces HSC output and can be rescued by endotome-derived-EC-specific expression of cxcl12b (Nguyen et al., 2014). Presumably, this function is cxcr4 dependent. Indeed another study in zebrafish showed that somite-derived retinoic acid signals induced cxcr4 expression on lateral plate mesoderm-derived HE during formation of the nascent aorta (Pillay et al., 2016). However, as CXCL12 can interact with other chemokine receptors and integrins (Balabanian et al., 2005; Burns et al., 2006), future studies will be required to determine the precise receptors CXCL12 interacts with to support HSC emergence from aortic HE. Taken together, these results intimate that HSC generation in the AGM is not solely dependent on EC/HE derived from the lateral plate mesoderm but also requires a unique somite-derived EC population that supports HSC development by contributing cell-extrinsic signals. Future studies are needed to further characterize the mechanism of CXCL12 function in HSC emergence, identify additional molecular signals that somite-derived ECs in the AGM may provide during HSC generation, and determine whether these roles are conserved in mammals.

### The Role of Extracellular Matrix and Integrins in HSC Development

Extracellular matrix proteins in the vascular niche may also contribute to HSC development. For example, hyaluronan (a.k.a. hyaluronic acid) was recently shown to influence hematopoietic development in the AGM via its receptor CD44. Although CD44 is known to influence homing and migration of hematopoietic stem/progenitor cells (HSPCs) to the bone marrow in adults (Schmits et al., 1997; Avigdor et al., 2004), its role in early HSC development in the AGM was only recently revealed (Oatley et al., 2020). Utilizing antibody screening and single-cell molecular analyses, it was discovered that CD44 expression is upregulated in a subset of arterial ECs transitioning from HE to HSCs and progenitors in the AGM. Interestingly, CD44 appears to be a driver, not simply a marker, of the endothelial to hematopoietic transition, as disruption of hyaluronan binding to CD44, via antibody blockage or inhibition of hyaluronan synthesis, reduces hematopoietic production from HE (Oatley et al., 2020). Although the source of hyaluronan production within the AGM is not yet defined, previous studies have shown that proinflammatory stimuli can regulate the endothelial cell production of hyaluronan *in vivo* and *in vitro* (Mohamadzadeh et al., 1998; Vigetti et al., 2010), suggesting that a similar mechanism may regulate its production within the AGM. Future studies of the downstream targets of CD44, the source(s) of hyaluronan, and the precise effect of hyaluronan–CD44 interactions on long-term engrafting HSCs will help further elucidate the mechanisms of this pathway on hematopoiesis in the AGM.

Integrins are a family of heterodimeric transmembrane glycoproteins composed of 18 α-type and eight β-type subunits that play important roles in numerous cellular processes by relaying intracellular signals via interactions with proteins in the extracellular matrix. Specific to the context of this review, integrins can facilitate cell adhesion events that are crucial to HSC development. In zebrafish, ECs derived from the lateral plate mesoderm migrate along the ventral side of the somites to establish the dorsal aorta. During this migration, ECs come in close contact with somitic cells expressing Dlc and Dld, which are orthologs of the mammalian Notch ligands Dll3 and Dll1, respectively (Kobayashi I. et al., 2014). When an interaction occurs, rap1b, the zebrafish ortholog to the small GTPase Rap1, can induce the activation of β_1_ integrin subunit (Itgb1) which promotes cell matrix adhesion by binding to fibronectin that accumulates at the somite boundary (Koshida et al., 2005). This enables sustained interactions between the Dlc/Dld ligands on somitic cells and Notch receptors on ECs, leading to the activation of Notch signaling required for the specification of HE (Rho et al., 2019). Thus, Itgb1-mediated adhesion promotes Notch-dependent HE specification within the dorsal aorta of zebrafish. Notably, rap1b mutants exhibit impaired HE specification that can be rescued by the overexpression of RIAM, which works downstream of Rap1/rap1b in integrin activation (Lafuente et al., 2004). This observation indicates that rap1b directly regulates Itgb1-mediated adhesion and raises the question of whether rap1b regulates other integrin-mediated events that influence HSC development (Satyanarayana et al., 2010; Rho et al., 2019).

The α_IIb_ integrin subunit (a.k.a. CD41) also appears to be important for HSC development. Indeed CD41 is among the earliest surface markers of the hematopoietic lineage (Mitjavila-Garcia et al., 2002; Mikkola et al., 2003) and is expressed by AGM HSCs and HSC precursors (Robin et al., 2011; Rybtsov et al., 2014). Live time-lapse imaging of mouse embryos revealed that CD41 is concomitantly expressed during the endothelial to hematopoietic transition in the aorta and that the protein localizes to points of contact between cells within intra-aortic hematopoietic clusters (Boisset et al., 2010, 2011). During this period, CD41 is believed to primarily form a co-receptor with β_3_ integrin subunit (a.k.a. CD61), which can interact with a variety of extracellular matrix proteins. However, despite its identity as an early hematopoietic marker, little is known about the functional role of CD41 during HSC development or the precise extracellular proteins with which it interacts in the vascular niche. Genetic ablation of CD41 results in hematopoietic defects (Boisset et al., 2013). However, these defects are subtle, suggesting a more nuanced role for CD41 and potential compensatory mechanisms mediated by other integrins expressed by HSCs and progenitors during their emergence from HE. Consistent with this hypothesis, the same study also found that CD51, another α integrin that can form a co-receptor with CD61, can partially compensate for the loss of CD41. Thus, future studies into the molecular mechanisms of CD41 and related integrins, including the identification of interacting proteins in the niche and downstream signaling pathways, will provide more insight into the functional role of integrins in HSC development.

### Other Niche Signals in HSC Development: Clues From AGM Stromal and Endothelial Cells

In addition to those described above, other signaling pathways activated by secreted cytokines and morphogens from a variety of sources, including niche stromal cells, influence the process of HSC production in the AGM. The notion that neighboring stromal cells provide support for HSCs dates back to when the term “stem cell niche” was first coined (Schofield, 1978). Historically, attempts to recreate an HSC niche have focused on the adult bone marrow stroma with the goal of promoting the *ex vivo* expansion of adult HSC. Similarly, studies of AGM-derived stromal cells have provided some insights into signals that promote HSC formation, maintenance, and expansion (Oostendorp et al., 2002a, b; Taoudi et al., 2008), which has translational importance for the goal of generating HSCs *de novo*, such as from pluripotent stem cells. Moreover, the ability to tightly regulate the environment of a coculture system, as compared to *in vivo*, has led to some key revelations in how niche cells influence hematopoietic development. In zebrafish, embryonic stromal trunk (ZEST) cells, derived from the tissues surrounding the embryonic dorsal aorta, were found to support both HSPC proliferation and multilineage differentiation *in vitro* (Campbell et al., 2015). ZEST cells express transcripts for several hematopoietic cytokines (kitl/SCF, fibroblast growth factor, interleukin-11, erythropoietin, *etc*.) as well as both Delta-like and Jagged-type Notch ligands. While ZEST cells were determined to have some characteristics of ECs, they also expressed markers of smooth muscle; thus, the exact *in vivo* stromal equivalent of ZEST cells in the zebrafish para-aortic tissues is unclear. Furthermore, while ZEST cells can support multilineage hematopoietic output from single embryonic CD41-expressing hematopoietic cells *in vitro*, it is not clear if ZEST stroma is sufficient to support HSC generation from resident HE, as it occurs in the aorta *in vivo*. In a stromal model consisting of ECs derived from murine AGM and transduced with constitutive AKT-expressing lentivirus to enable their survival in serum-free conditions, generation and expansion of long-term engrafting HSCs from embryonic HE were demonstrated *in vitro* (Hadland et al., 2015). Further analysis of this coculture system revealed that the AGM-derived EC support of HSC development from early HE (i.e., VE-cadherin^+^ cells isolated from murine P-Sp/AGM as early as E9), as well as the subsequent expansion of HSCs *in vitro*, is Notch dependent. While AGM-derived ECs were shown to express multiple Notch ligands (including Dll4, Dll1, and Jagged1), further studies are needed to understand the dynamics of Notch signaling and the role of other niche factors expressed by AGM-derived ECs during HSC formation in this *ex vivo* system.

While these studies using AGM-derived ECs and stromal cells lay the foundation for identifying the signals necessary to support the *in vitro* specification and self-renewal of HSCs, further optimization of engineered stromal niches is needed to evaluate the influence of factors like temporal modulation of key signal pathways, biomechanical properties, and 3D structure.

A recent study utilized RNA tomograph (tomo-seq), a powerful approach that enables the generation of a genome-wide atlas of gene expression for a specific tissue, to explore the AGM microenvironment across species (Yvernogeau et al., 2020). Specifically, the authors were able to identify two novel pathways important for HSC formation and conserved across human, mouse, chicken, and zebrafish development. The first involves the ligand ADM (adrenomedullin), which was found to be expressed in the AGM microenvironment, and its receptor RAMP2, which is expressed by aortic endothelium and HSPCs. Subsequent experiments of *in vivo* chick and zebrafish models as well as *ex vivo* mouse models revealed that depletion of ADM, either genetically or chemically, reduced HSPC production in all model systems. The second factor, SVEP1, which has previously been shown to regulate the sprouting and the migration of venous endothelial cells (Karpanen et al., 2017), is secreted into the extracellular matrix, most likely by mesenchymal cells surrounding HSPCs. Loss of SVEP1 secretion in both mouse and zebrafish embryos results in a marked decrease in HSPC production, yet it does not completely abrogate the long-term engraftment capacity in the mouse model (Yvernogeau et al., 2020). Although future studies are needed to precisely identify the cells responsible for producing ADM and SVEP1 and further examine the downstream pathways and transcriptional programs regulated by these factors, this study highlights a powerful approach that may be able to identify additional unknown pathways and factors that are important for HSC development and conserved across species.

### Contribution to HSC Development by Other Niche Cells in the AGM

In addition to endothelial and stromal cells in the AGM, a plethora of different cell types, including some that migrate to the AGM region from other tissues, also contribute to HSC development in the AGM vascular niche.

One of the more intriguing examples of this is yolk sac-derived macrophages that migrate to the AGM (Mariani et al., 2019). This migration is facilitated by the chemokine receptor Cx3cr1 and occurs immediately prior to and continues through the initial emergence of HSC within the AGM (E9-11). Upon arrival, the yolk sac-derived macrophages directly interact with emerging intra-aortic hematopoietic clusters and promote a pro-inflammatory response within the AGM microenvironment to support HSC induction (Mirantes et al., 2014). In contrast, studies in zebrafish suggest that neutrophils derived from the EMP wave are thought to be the primary source of proinflammatory cytokines around the dorsal aorta, inducing HSC development by activating the Notch pathway downstream of TNF-alpha-induced Jagged1 expression (Espín-Palazón et al., 2014). Future studies will be required to determine if these findings demonstrate overlapping roles for distinct lineages of yolk sac-derived myeloid progeny in promoting HSC development or if they reflect species-specific differences.

The sympathetic nervous system has also been shown to play a role in HSC emergence in the AGM (Fitch et al., 2012), driven by catecholamine production from Gata3-expressing sympathoadrenal cells located in two lateral patches on either side of the dorsal aorta. Interestingly, nascent HSCs within emerging intra-aortic hematopoietic clusters express the β_2_-adrenergic receptor (Adrb2), thus making them receptive to catecholamines. Reduced catecholamine production (via the genetic ablation of *Gata3* or *Th*, which encodes tyrosine hydroxylase, an enzyme required for catecholamine biosynthesis, or by pharmacological inhibition) leads to a marked reduction in HSC numbers and function within the AGM. Moreover, this defect is independent of any hemodynamic forces mediated by catecholamines (Zhou et al., 1995). Further characterization of the transcriptional programs regulated by catecholamine-mediated Adrb2 activation in emerging HSCs will provide insight into how HSC development is regulated by this mechanism.

### Hemodynamic Forces in HSC Development

One of the more intriguing, but also ill-defined, aspects of the arterial vascular niches in which HSCs emerge are the hemodynamics forces at play. Accumulating evidence suggests that these forces may have a profound impact on hematopoietic specification and activity in the P-Sp/AGM, where several factors including merging of the paired dorsal aorta, initiation of the heartbeat, and dynamically increasing pulsatile blood flow converge to influence the biomechanical properties of the aorta (Adamo et al., 2009; North et al., 2009; Kim et al., 2015; Fang et al., 2017; Lundin et al., 2020). Several types of hemodynamic forces are at play due to the pulsatile nature of blood flow, including circumferential stress, hydrodynamic pressure, and fluid shear stress (Figure 1). Fluid shear stress, which is frictional force on the surface of cells lining a vessel generated by the flowing blood, is the most well-studied in the context of hematopoietic activity and has been found to be critical for proper HSC development in the AGM (Adamo et al., 2009; Wang et al., 2011), though recent studies also demonstrate a distinct role for circumferential forces through cyclic stretch in HSC formation (Lundin et al., 2020).

The influence of fluid shear stress on hematopoietic activity was described by Adamo and colleagues when they observed that differentiated murine ESCs exposed to fluid shear stress exhibited increased hematopoietic progenitor output and expression of the hematopoietic transcription factor *Runx1* (Adamo et al., 2009). They further determined that this phenomenon occurred *in vivo* and that nitric oxide (NO) signaling, a pathway well-known to be regulated by fluid shear stress (Garcia-Cardena et al., 1998), influenced hematopoietic activity. A similar role for NO signaling downstream of fluid shear stress in HSPC formation in zebrafish suggests a highly conserved mechanism (North et al., 2009). It has since been shown that kruppel-like transcription factor 2 (*klf2*), a known early responder to blood flow dynamics (Lee et al., 2006), acts upstream of NO signaling to regulate artery maturation and hematopoietic programming within the AGM. Specifically, klf2 promotes NO signaling by binding to and transcriptionally activating NO pathway members *nos1* and *nos2b*, which ultimately results in the increased expression of *runx1*. Moreover, *klf2*-MO embryos, which exhibit reduced HSC numbers and runx1 expression, can be rescued by the addition of the NO agonist, SNAP (Wang et al., 2011). This observation further underscores the notion that blood flow mediates HSC development in the AGM through a Klf2–NO axis. However, the precise mechanism, direct or indirect, by which NO signaling regulates runx1 expression remains uncharacterized. Other signaling pathways involved in HSC development have also been shown to be influenced by fluid shear stress within the context of the AGM (Kim et al., 2015; Fang et al., 2017). For example, protein kinase A (Pka) and its downstream target cAMP response element-binding protein (Creb) are activated by exogenous fluid shear stress and interact with BMP signaling factors Smad1 and its co-factor E2f4 (Kim et al., 2015). These interactions lead to the downstream expression of hematopoietic factors like Gata2 and Gata3 as well as the adult HSC maintenance factor, Pbx1 (Ficara et al., 2008). Interestingly, at the onset of circulation, both hemogenic and non-hemogenic endothelium are sensitive to this PKA–CREB–BMP signaling axis, whereas progenitor cells are not (Kim et al., 2015). This observation suggests that cells must be actively expressing endothelial programs in order to be influenced by this pathway and that the activation of PKA–CREB–BMP signaling facilitates the transition from an endothelial to a hematopoietic program.

Intriguingly, Notch activity is also sensitive to fluid shear stress, leading to the upregulation of the arterial endothelial gene *Gja4* (Cx37) and its downstream target *Cdkn1b* (p27) (Fang et al., 2017). Cdkn1b-depedent cell cycle arrest upregulates additional arterial markers like *Efnb2* and *Gja5* and promotes arterial specification. Notably, the depletion of either *Gja4* or *Cdkn1b* abrogated *Efnb2* and *Gja5* expression in the context of fluid shear stress and Dll4-induced Notch activity, indicating that Gja4 and Cdkn1b function downstream of Notch in activating arterial gene programs. The activation of Cdkn1b has also previously been implicated in triggering G1 phase cell cycle arrest during HE specification downstream of Notch signaling (Marcelo et al., 2013), but whether Notch acts as a mechanosensor within the context of HSC development is unknown; if true, this mechanism could be exploited to regulate dynamic Notch signaling activation for the optimization of HSC development *in vitro*.

As previously mentioned, fluid shear stress is not the only hemodynamic force that can influence HSC development. Blood flow-associated cyclic stretch, which is the outward pressure put on cells lining blood vessels, can also regulate HSC development. Specifically, exposure of human pluripotent stem cell-derived HE to cyclic stretch was observed to stimulate YAP signaling in a Rho-GTPase-dependent manner. *In vivo* modeling in zebrafish revealed that upregulation of yap1 activity helps to maintain the hematopoietic program in HE by promoting the expression of *runx1* and *cmyb* (Lundin et al., 2020). This regulatory mechanism is specifically sensitive to cyclic stretch, as *in vitro* exposure to fluid shear stress did not elicit the same physiologically relevant stimulation of YAP signaling within the context of HSPC production. Interestingly, there is also evidence for crosstalk between YAP and Notch signaling during different stages of development in the context of other tissues (Totaro et al., 2018). Thus, it will be interesting to see how the interplay between these two pathways may influence HSC development. Another receptor/pathway to note for future studies is Piezo1, which has been shown to be critical for vascular development and is sensitive to fluid shear stress and other biomechanical forces such as vessel stretching (Ranade et al., 2014; Hyman et al., 2017). Ultimately, future investigations will shed light as to how multiple hemodynamic forces, including fluid shear stress, cyclic stretch, and blood pressure, dynamically regulate various pathways to affect different stages and aspects of HSC formation within the aorta and other arterial vessels during embryonic development.

### HSC Generation and Expansion in Other Embryonic Vascular Niches

Although the emergence of HSCs has been most extensively characterized in the context of the dorsal aorta of the AGM, HSCs are also independently generated from HE in extra-embryonic arteries (but not veins), including the vitelline artery connecting to the yolk sac and the umbilical artery connecting to the placenta (de Bruijn et al., 2000; Gordon-Keylock et al., 2013). The placenta has also been identified as a vascular niche for both *de novo* hematopoiesis as well as transient expansion of HSC activity (Gekas et al., 2005, 2010; Ottersbach and Dzierzak, 2005). A diverse repertoire of niche cells, including ECs, pericytes, mesenchymal stromal cells, and trophoblasts, may contribute to the placental hematopoietic niche by providing hematopoietic cytokines and other supportive factors. Interestingly, whereas HSCs and hematopoietic progenitors are generated in association with the large placental vessels, expansion of HSCs occurs distinctly in smaller labyrinth vessels of the placenta, suggesting that this site may offer clues about unique niche hematopoietic factors required for HSC self-renewal (Gekas et al., 2010). Recently, the cerebrovascular endothelium of the embryonic head and arterial-like endothelium in the fetal bone marrow have also been described as sites of *de novo* HSC generation (Li et al., 2012; Yvernogeau et al., 2019). Future studies to investigate the overlapping vascular niche signals expressed in these various locations may provide important insights into the core signals required for HSC genesis.

## HSC Expansion and Hematopoietic Progenitor Differentiation in the Fetal Liver Vascular Niche

### Colonization of the Fetal Liver With HSCs and Progenitors

In the above sections, “HSC-Independent Hematopoietic Progenitors in the Yolk Sac Vascular Niche” and “HSC Development in the Arterial Vascular Niche”, we reviewed the role of the vascular niche in regulating the development of HSCs and earlier waves of HSC-independent hematopoietic progenitors. Following their formation from HE in the blood vessels of the yolk sac, AGM, and other tissues, the newly emerging HSCs and progenitors enter circulation to colonize the fetal liver, where they undergo further maturation, expansion, and differentiation. To this end, the fetal liver is initially seeded by HSC-independent progenitors, which undergo further proliferation and rapid differentiation to blood cells which supply the needs of the developing embryo (Mikkola and Orkin, 2006; Papathanasiou et al., 2009). The initial HSCs that have formed in the AGM by E11 are heterogenous in their repopulating ability, demonstrated by an infrequent, long-term, multilineage engraftment capacity, with a large portion of immature “pre-HSC” that are further matured to long-term engrafting HSCs during and/or following their colonization of the fetal liver between E11 and E12 (Kieusseian et al., 2012; Rybtsov et al., 2016). Furthermore, HSCs also undergo significant numerical expansion by proliferation in the fetal liver (Ohneda and Bautch, 1997; Mikkola and Orkin, 2006; Papathanasiou et al., 2009; Poisson et al., 2017). Following maturation and expansion in the fetal liver, HSCs migrate to the bone marrow during late gestation. Given that the fetal liver serves such diverse roles during hematopoietic development, much can be learned about the various aspects of hematopoiesis from studying the contributions of the heterogeneous microenvironmental compartments in the fetal liver. Furthermore, as ECs within different organs demonstrate distinct gene expression patterns and production of local niche factors to influence the development of tissue-specific stem/progenitor cells, it is likely that the detailed study of fetal liver vascular niche heterogeneity will be important to understand the signaling pathways that regulate various hematopoietic fates (Rafii et al., 2016; Marcu et al., 2018). In this regard, many recent elegant studies have revealed new insights into the fetal liver vascular niche and the dynamic changes during fetal liver development that contribute to regulating HSC and progenitor fates. Here we summarize the latest understanding of how hematopoiesis is regulated in the mammalian fetal liver vascular niche and the caudal hematopoietic tissue (CHT) in zebrafish, a vascular plexus in the ventral tail of the embryo which is thought to serve an equivalent role in hematopoiesis to the mammalian fetal liver.

### Mechanical Interactions Between CHT/Fetal Liver ECs and Self-Renewing HSCs

The zebrafish model has been a powerful tool for uncovering the biological mechanisms of hematopoietic development. As zebrafish embryos are transparent, developmental events such as HSC emergence, migration, and engraftment can be monitored non-invasively *in vivo*, providing visual clarity into the vascular niche. In zebrafish, HSPCs emerge in the dorsal aorta around 30 hours post-fertilization (hpf), circulate to the CHT around 36 hpf, and then ultimately colonize the thymus and kidney, the adult stem cell niches in zebrafish, at 4 days post-fertilization (Murayama et al., 2006; Bertrand et al., 2010; Kissa and Herbomel, 2010; Tamplin et al., 2015; Perlin et al., 2017a, b).

**FIGURE 2:**
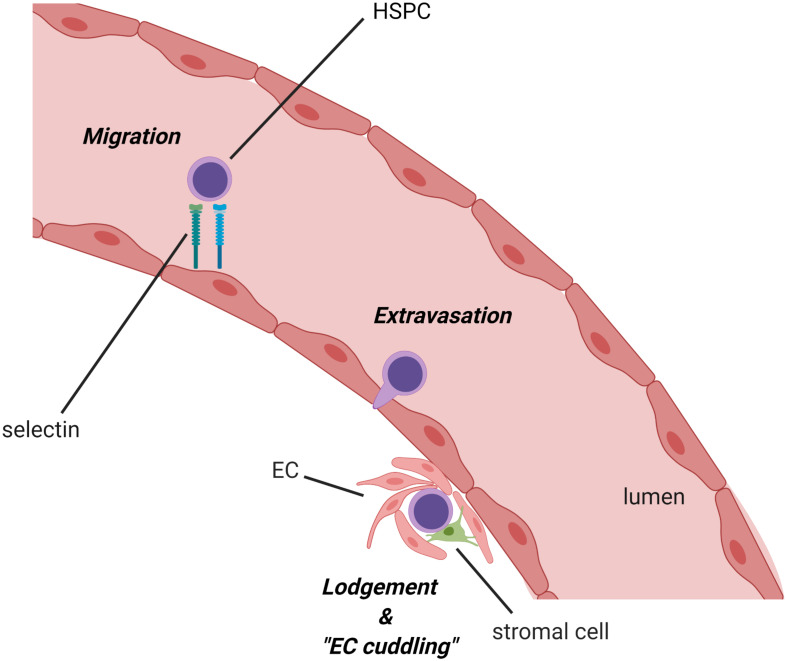
Migration of hematopoietic stem/progenitor cell (HSPC) into the zebrafish caudal hematopoietic territory (CHT) and remodeling of the ECs (EC cuddling). Following their emergence in the dorsal aorta, HSPCs circulate to the CHT via the caudal artery, adhere to the endothelial cell wall, infiltrate across the vessel, and lodge in the niche. Once HSPCs are embedded in the niche, nearby ECs remodel around the HSPCs to form a pocket in a process referred to as “endothelial cell cuddling.” This process, which appears to be conserved in the mammalian fetal liver as well, facilitates the interaction of HSPCs with surrounding niche cells, such as sinusoidal ECs and stromal cells, which provide supportive niche factors for the survival, self-renewal, and differentiation of HSPCs to influence diverse hematopoietic fates. HSC, hematopoietic stem cell; EC, endothelial cell (created with BioRender.com).

It is well-established that the fetal liver niche orchestrates HSC proliferative expansion during development, but the mechanisms regulating this process, particularly those involving interactions between HSCs and ECs or other fetal liver stromal cells in the niche, have been largely unresolved. However, studies of the zebrafish CHT have provided useful insights. For example, Tamplin and colleagues used a specific transgenic reporter for the detection of *Runx1*-expressing HSPCs in zebrafish to enable high-resolution live imaging of the interactions between single HSPC and cells in the CHT niche (Tamplin et al., 2015). Strikingly, when HSPCs migrate into the CHT through circulation and infiltrate across the vessel lumen to colonize the perivascular niche, a group of ECs remodel to form a surrounding pocket. This remodeling of the ECs, termed “EC cuddling,” is initiated by the arrival of HSPCs and promotes contact of HSPCs with other surrounding supporting cells such as stromal cells and fibroblasts (Perlin et al., 2017b) (Figure 2). Subsequently, the mesenchymal stromal cells anchor HSPCs, promoting HSPC division and expansion in the CHT niche. A total of 60% of HSPCs in the CHT are directly adjacent to cxcl12a-expressing stromal cells, and 85% are within a 3-μm distance. Notably, time-lapse analysis unveiled that the arrival and expansion of HSPCs in the CHT are specifically captured within the region where the stromal cells are closely attached. Furthermore, HSPC cell division in this specialized niche is asymmetric as the daughter cell proximal to the cxcl12a^+^ stromal cells remains in the niche, while the daughter cell distal to the stromal cells moves away from the niche. This finding suggests that the adjacent stromal cells may relay a distinct signal that initiates the asymmetrical division of HSPCs. Further analysis by live imaging in E11.5 murine fetal liver explants showed that this structure was conserved in mouse. Importantly, a chemical genetic screen identified the compound lycorine (a natural alkaloid extracted from the Amaryllidaceae plant family) could promote HSPC–niche interactions in the CHT, leading to a sustained increase in the HSPC pool in the adult kidney marrow (Tamplin et al., 2015). While the exact mechanism of lycorine’s effect on HSPC expansion in the CHT is yet to be determined, lycorine has been shown to exert anti-inflammatory and anti-tumor effects (Kang et al., 2012). Consistent with this potential mechanism, whole genome microarray analysis of runx1^+^ HSPCs and kdrl^+^ ECs after lycorine treatment identified changes in the adhesive properties of ECs and the activation states of HSPCs, which could be related to the effects of lycorine on inflammatory pathways.

### EC and HSC Interactions Regulate HSC Expansion in the CHT/Fetal Liver Niche

CHT ECs are reported to express many cytokines underlying HSPC expansion and differentiation such as Kit ligand (KitL, also known as SCF), thrombopoietin, oncostatin M, and colony-stimulating factor 3a. Particularly, KitL and oncostatin M synergistically enhance HSPC expansion and inhibit lymphoid differentiation (Mahony et al., 2018). Recently, lipoprotein lipase (lpl) expressed in CHT ECs was also reported to be a novel supportive factor for HSPC expansion in the CHT niche (Liu et al., 2018). Lpl and its obligatory cofactor apolipoprotein c2 (apo2) regulate the release of the essential free fatty acid (FFA) docosahexaenoic acid (DHA). Exogenous FFA-DHA enhanced the expression of hematopoietic genes *runx1*, *cmyb*, and *beta-globin* in wild-type mice and furthermore restored the expression of these genes in *lpl* and *apoc2* mutant zebrafish. While the exact mechanism of how FFA-DHA regulates the maintenance and expansion of HSPCs remains to be elucidated, this study suggests an important role for ECs in the CHT vascular niche in supporting the metabolism of developing fetal HSPCs.

Recently, signaling between the chemokine cxcl8 and its receptor, cxcr1, both expressed by ECs in the CHT niche, was shown to facilitate EC cuddling and, ultimately, the proliferation of HSPCs (Blaser et al., 2017). In addition to ECs, the source of cxcl8 is also speculated to be from myeloid cells or even HSPCs in the CHT niche (Zhao et al., 2014). Mechanistically, cxcl8/cxcr1 signaling induces the EC expression of vegfa and survivin in an autocrine and/or paracrine fashion, thus enhancing the survival and motility of sinusoidal ECs in the CHT. In turn, this increases the overall volume of the CHT niche and enhances the expression of cxcl12a/sdf-1a, resulting in the retention of HSPCs, enhanced EC cuddling, and increased residency time within the CHT niche (Blaser et al., 2017). Remarkably, overexpression of cxcr1 in the recipient niche accelerated the engraftment of HSPCs when tested in a parabiotic zebrafish model (Blaser et al., 2017). How this process is terminated and how HSPCs are triggered to exit the CHT vascular niche later in development when they circulate to the kidney marrow, which serves at the site of adult hematopoiesis in the zebrafish, remain to be determined.

In the murine fetal liver, a recent study demonstrated a role for adhesive interactions between periostin, expressed on fetal liver ECs, and αv-integrin, expressed on HSCs, in regulating the size of the fetal HSC pool by limiting proliferation (Biswas et al., 2020). Interestingly, the genetic deletion of periostin in fetal liver ECs enhanced the proliferation of fetal liver stage HSCs that maintained their long-term engraftment properties as assayed by serial transplantation studies. In contrast, previous studies had shown that the loss of periostin–integrin interactions in adult bone marrow HSCs caused the proliferation of HSCs with concomitant loss of long-term engraftment potential (Khurana et al., 2016). These contrasting findings suggest potential cell-intrinsic differences between fetal liver and adult bone marrow HSCs, which are hypothesized to be linked to differences in DNA repair programs, although the influence of other niche-specific factors has not been ruled out. Together with the above-mentioned studies, these findings suggest that ECs, through direct interactions with HSCs in the fetal liver/CHT niche, may tightly regulate the expansion of the HSC pool by providing both supportive and inhibitory signals to HSC proliferation during fetal development.

### Transcription Factors Confer HSC-Supportive Properties to Fetal Liver/CHT Niche ECs

Following the seeding of HSCs to the fetal liver/CHT, HSCs experience significant proliferation within the fetal liver/CHT niche. As described in the previous sections, “Mechanical Interactions Between CHT/Fetal Liver ECs and Self-Renewing HSCs” and “EC and HSC Interactions Regulate HSC Expansion in the CHT/Fetal Liver Niche”, the retention of HSCs in the CHT vascular niche is required for this expansion and is regulated through chemokines and other niche factors derived from the CHT stroma, particularly ECs. Building on this work, several studies have highlighted the role of transcription factors in conferring ECs within the fetal liver/CHT niche with their unique HSC-supportive properties.

The transcriptional factor Kruppel-like factor 6a (*klf6a*) was recently shown to control the recruitment and expansion of HSPCs in the CHT niche (Xue et al., 2017). Specifically, *klf6a*-deficient embryos exhibited decreased HSPCs in the CHT, secondary to defects of lodgment and proliferation. Transplantation of HSPCs from *klf6a*-deficient embryos into wild-type embryos and reciprocal transplantation of wild-type HSPCs into *klf6a*-deficient embryos suggested that a cell-extrinsic defect in the CHT environment is responsible for HSPC impairment in the absence of *klf6a*. Mechanistically, klf6a directly regulates the expression of chemokine ligand 25b (ccl25b, the homolog of Ccl21 in mammals). ccl25b is expressed in zebrafish caudal vein ECs (Lu et al., 2012) and binds to its cognate receptor C-C motif receptor7 (CCR7) expressed on HSPCs, thus promoting the migration of HSPCs and their proliferation in the caudal venous plexus of the CHT. In the murine fetal liver at E14.5, Klf6 and Ccl21 were enriched in ECs, while Ccr7 was highly expressed in ckit^+^sca1^+^Lineage^–^ HSPCs. Fetal liver ckit^+^sca1^+^Lineage^–^ cells cultured in the presence of Ccl21 demonstrated enhanced colony-forming capacity with multilineage differentiation. Moreover, this effect was abrogated by the blockade of Ccl21, suggesting that the pathway is conserved in mammals (Xue et al., 2017).

Tfec, a basic helix-loop-helix transcription factor that belongs to microphthalmia-associated transcription factor family member, is also highly enriched within ECs in the CHT niche (Mahony et al., 2016). Overexpression of tfec increases the number of HSPCs in the CHT, whereas embryos lacking tfec exhibit loss of HSPCs after emergence from the ventral dorsal aorta. Tfec regulates expansion of HSPCs by modulating the expression of cytokines including KitL, thrombopoietin, and colony-stimulating factors 1a and 3b. Among these, the overexpression of KitL rescued tfec mutants. Overall, tfec mediates HSPC proliferation and expansion by modulating cytokine expression from CHT ECs.

In the murine model, sinusoidal EC-restricted deletion of Gata4 resulted in defective sinusoidal EC formation, hypoplasia and fibrosis of the fetal liver, and impaired colonization by HSPCs (Géraud et al., 2017). These defects lead to severe anemia and embryonic lethality, suggesting a requirement for Gata4 in the development of fetal liver ECs that critically support developing HSPCs in fetal liver. Activating transcription factor (Atf4) also plays in important role in the expansion and the maintenance of resident HSPCs in the fetal liver by upregulating angiopoietin-like protein 3, a cytokine known to promote HSPC expansion (Zhang et al., 2006), in fetal liver ECs and stromal cells (Zhao et al., 2015).

Interestingly, epigenetic regulators, like polycomb-group proteins which mediate chromatin modifications, can also influence hematopoiesis through cell-extrinsic effects on the fetal liver vascular niche. For example, targeted endothelial-specific deletion of Ezh2, a core component of polycomb repressive complex 2, results in lethal anemia of murine embryos at E13.5, while HSCs initially emerge normally (Neo et al., 2018). Mechanistically, the inactivation of Ezh2 in fetal liver ECs leads to the overexpression of matrix metalloprotease 9 (Mmp9), which cleaves the membrane-bound form of KitL (mKitL), a crucial factor for fetal liver erythropoiesis. Inhibition of Mmp9 *in vitro* restored the erythropoiesis-supportive capacity of fetal liver ECs by maintaining mKitL expression. Thus, these results suggest that the expression of Ezh2 in fetal liver ECs mediates fetal erythropoiesis through SCF/mKitL signaling.

Collectively, these studies highlight a prominent role for transcription factors and epigenetic modulators as master regulators of ECs in the fetal liver/CHT niche, imparting these niche ECs with unique properties essential for their ability to support various aspects of HSPC expansion and differentiation to hematopoietic lineages during development. Future studies will be required to further understand how these various transcription factors, and others yet undiscovered, coordinate gene expression programs to specify subsets of fetal liver ECs that may support different hematopoietic processes, such as the expansion of HSCs versus lineage-specific differentiation from progenitors.

### Contribution of Dynamic Blood Flow Changes Between Fetal and Post-natal Development to HSC Biology and Mobilization From Fetal Liver to Bone Marrow

While the studies mentioned above establish a role for biochemical signals provided by fetal liver/CHT vascular niche ECs in supporting HSC expansion, the impact of the vascular architecture and the biomechanical forces of blood flow in the fetal liver on HSC development has not been as extensively studied. Recently, however, Khan and colleagues demonstrated that the unique hemodynamics of circulation in the fetal liver plays an important role in regulating the HSC-supportive properties of the vascular niche during the pre-natal and the post-natal period in the mouse model (Khan et al., 2016). Specifically, in the context of fetal circulation, blood flow from the placenta via the portal vein exposes ECs within the hepatic portal vessels to relatively high pressure, and these ECs exhibit an arterial-like phenotype. The portal vessels are closely associated with Nestin^+^NG2^+^ pericytes, which also express arterial endothelial markers such as ephrin-B2 and neuropilin-1 and are enriched with HSC-supportive niche factors such as SCF, angiopoietin II, Igf2, and Cxcl12. The Nestin^+^NG2^+^ pericytes are highly proliferative, significantly expanding from E12 to E14.5, concomitant with the increasing surface area of the portal vessels in a fractal branching pattern. This, in turn, parallels the expansion of HSCs, which localize closely to Nestin^+^NG2^+^ pericytes on portal vessels, suggesting that these cells may serve a supportive role in HSC expansion in the fetal liver vascular niche. Interestingly, when the umbilical vein closes at birth, the features of ECs in the portal vessels dramatically change in association with the resulting lower-pressure hemodynamics of post-natal circulation in the portal vein. Specifically, ECs of the portal vessels transition from a neuropilin-1^+^ ephrin-B2^+^ arterial phenotype to an EphB4^+^ venous phenotype, accompanied by a loss of periportal Nestin^+^NG2^+^ cells and extravasation of HSCs away from the portal vessels. Altogether, these studies suggest a novel mechanism that ties the hemodynamic state of the fetal liver vascular niche to its HSC-supportive functions and provide new insights into the mechanisms regulating the transition of HSCs from the fetal liver to the bone marrow that occurs around the time of birth. Future studies will be required to further elucidate the molecular signals that link blood flow dynamics to EC and pericyte phenotype and function in the fetal liver HSC-supportive vascular niche and to elucidate the precise niche factors necessary for fetal HSC expansion in this context. This study also raises several important questions. For one, as opposed to previous studies of ECs in the CHT/fetal liver niche, which have largely characterized hematopoietic-supportive ECs as sinusoidal, the current study suggests that arteriolar-like ECs in the portal vessels, and a closely associated population of periportal pericytes, serve as the primary niche for HSC expansion in the fetal liver. Thus, additional studies will be needed to further clarify whether different hematopoietic niches in the fetal liver can have overlapping functions in HSC expansion and/or whether different fetal liver compartments play distinct roles in the recruitment, maturation, expansion, and differentiation of various subsets of HSCs and progenitors as they asynchronously colonize the fetal liver. Particular attention to the impact on functional HSC output, as measured by clonal lineage tracing *in vivo* or long-term multilineage engraftment in transplantation assays, will be important in future investigations, as many previous studies have evaluated the effects on populations of HSPCs based on phenotypic markers, reporter gene expression, or *in vitro* hematopoietic potential that may not be specific to bone fide HSCs.

Altogether the studies reviewed in this section in both murine and zebrafish models provide novel mechanistic insights into how the CHT/fetal liver vascular niche regulates the lodgment, expansion, and differentiation of HSCs and progenitors during fetal development. Importantly, growing knowledge of the unique niche factors expressed by ECs and other CHT/fetal liver stromal cells during their interactions with HSCs could have important translational applications to enable HSC expansion *ex vivo*, broadening the availability of and improving clinical outcomes following hematopoietic cell transplantation.

## Conclusion and Future Perspectives

Hematopoiesis in the embryo is an inherently dynamic process as numerous objectives, including the swift production of diverse, functioning blood cells and the creation of self-renewing HSCs, must be carried out in a timely, sequential, and organized fashion to meet the needs of the growing organism while also providing a stem cell pool for life-long hematopoiesis. In this review, we have described how specialization of the vascular niche, in the context of a variety of organs and tissues throughout development, plays a central role by orchestrating the sequential waves of developmental hematopoiesis necessary to carry out these diverse processes. Although a comprehensive review of the large and growing body of literature in this field is prohibitive, we have focused on studies highlighting several central and recurring concepts, as well as recent studies providing provocative new insights which open up novel questions for future exploration and offer pathways to clinical translation. To this end, one key mechanism that has emerged from numerous studies, particularly in the context of the earliest waves of HSC-independent hematopoiesis, is how multiple cell extrinsic signals from the vascular niche, including inhibitory or feedback signals, are integrated with the cell intrinsic properties of hemogenic precursors to control the rapid and transitory emergence of hematopoietic progenitors. This is essential to generate bursts of hematopoietic activity as they are precisely needed during development, such as the initial production of the first circulating primitive erythrocytes in the yolk sac blood islands necessary for oxygen delivery at the onset of circulation. Given that some HSC-independent progeny, such as tissue resident macrophages and innate B1a-lymphocytes, persist into adulthood with functions in tissue homeostasis and immunity, a deeper understanding of how these cells develop and how they could potentially be engineered from pluripotent stem cells may be of clinical significance. Another key insight has come from recent studies that have demonstrated an important function for arterial endothelial specialization in the vascular niche during the emergence of multilineage hematopoiesis and HSCs. Several core pathways, including Notch and Wnt, as well as various hemodynamic forces induced by the onset of blood flow, have been shown to be essential in this process, and furthermore must be dynamically regulated during the sequential formation of arterialized HE and subsequent transition to functional HSCs in arterial vessels. How these pathways integrate, however, to orchestrate HSC development remains unclear. Particularly, understanding the signals that must uniquely converge to generate self-renewing HSCs, as opposed to LMPs and other multilineage hematopoietic progenitors that also arise from arterial HE, is a priority topic for further investigation given that this knowledge is critical to long-standing efforts to engineer HSCs *de novo* from pluripotent stem cells for clinical applications. Finally, transcriptional regulation of EC specialization in the CHT/fetal liver niche is another emerging area of active investigation. While a number of transcriptional and epigenetic regulators of the HSC-supportive properties of ECs have recently been described, how these factors contribute to EC niche heterogeneity, and in turn to the diverse functions of the fetal liver, including maturation and expansion of HSCs, and lineage-specific differentiation of hematopoietic progenitors, remains to be thoroughly explored. Given the unparalleled HSC expansion that occurs uniquely in the context of the fetal liver, this knowledge could also have profound clinical applications toward the goal of engineering methods to expand HSCs *ex vivo*, to improve the efficacy of gene therapy and hematopoietic cell transplantation critical for the treatment of blood and immune disorders.

## Author Contributions

All authors listed have made a substantial, direct and intellectual contribution to the work, and approved it for publication.

## Conflict of Interest

The authors declare that the research was conducted in the absence of any commercial or financial relationships that could be construed as a potential conflict of interest.
